# Improving Diagnostic Workup Following Traumatic Spinal Cord Injury: Advances in Biomarkers

**DOI:** 10.1007/s11910-021-01134-x

**Published:** 2021-07-16

**Authors:** Simon Schading, Tim M. Emmenegger, Patrick Freund

**Affiliations:** Spinal Cord Injury Centre, Balgrist University Hospital, University of Zurich, Forchstrasse 340, 8008 Zurich, Switzerland

**Keywords:** Spinal cord injury, Biomarker, Severity assessment, Prognostic factors, Pathophysiology, Predictive model

## Abstract

**Purpose of Review:**

Traumatic spinal cord injury (SCI) is a life-changing event with drastic implications for patients due to sensorimotor impairment and autonomous dysfunction. Current clinical evaluations focus on the assessment of injury level and severity using standardized neurological examinations. However, they fail to predict individual trajectories of recovery, which highlights the need for the development of advanced diagnostics. This narrative review identifies recent advances in the search of clinically relevant biomarkers in the field of SCI.

**Recent Findings:**

Advanced neuroimaging and molecular biomarkers sensitive to the disease processes initiated by the SCI have been identified. These biomarkers range from advanced neuroimaging techniques, neurophysiological readouts, and molecular biomarkers identifying the concentrations of several proteins in blood and CSF samples. Some of these biomarkers improve current prediction models based on clinical readouts. Validation with larger patient cohorts is warranted.

**Summary:**

Several biomarkers have been identified—ranging from imaging to molecular markers—that could serve as advanced diagnostic and hence supplement current clinical assessments.

## Introduction

Traumatic spinal cord injury (tSCI) is a devastating event and most often leads to serious sensorimotor deficits and autonomous dysfunctions. The worldwide yearly incidence of SCI is estimated at 40 up to 80 cases per million [1]. While traumatic causes such as road traffic incidents and falls are the predominant etiology in the younger population, non-traumatic causes, including neoplastic tumors and degenerative conditions, increase with age. Currently there is no cure, and functional recovery is limited, leaving the majority of patients with severe and permanent impairments [2]. Accordingly, patients are eager to know their prognosis soon after the injury, as to whether they will regain voluntary control of upper and lower limbs as well as autonomous function [3]. Therefore, advanced diagnostic measures that can increase the accuracy of prediction models which ultimatively can predict individual trajectories of recovery are required.

Routine clinical evaluations for assessing the current clinical impairment include performing the International Standards for the Neurological Classification of Spinal Cord Injury (ISNCSCI) protocol at admission. Based on this assessment, the patient’s overall impairment is classified on the American Spinal Injury Association (ASIA) Impairment scale (AIS) [4, 5]. This scale represents the gold standard assessment for documentation of the level and severity of a SCI [5]. It classifies patients according to their motor and sensory impairment into five categories (A–E) with category A corresponding to the most severely impaired patients and category E indicating no clinically relevant impairment. A recent review analyzing multiple clinical and demographic factors and their contribution to the prediction of global functional outcome concluded that the severity of SCI measured by the AIS was the strongest predictive factor [6]. However, the major limiting factor for using the AIS scores for prediction is the substantial heterogeneity of individual recovery potentials in each category. This may lead to a diverse range of recovery trajectories between patients with similar initial clinical impairment and result in different long-term functional outcomes, which highlights the need for more refined assessments and the incomplete understanding of the exact pathophysiological mechanisms [7, 8]. Moreover, differences in neurological examination timing introduce additional heterogeneity in the classification of patients due to higher variability in spontaneous recovery during the early phase after injury. Therefore, outcome prediction based on neurological assessments performed very early after injury may differ from predictions based on neurological examinations at later stages [9]. Another drawback arises in cases of intoxication, sedation, or patients with concomitant brain injuries, which makes the initial neurological assessment challenging [10•, 11]. Hence, advanced diagnostics which can describe the impact of the trauma independent from the neurological assessment are required. This narrative review critically evaluates the literature from the past 5 years and provides an overview of the developments in identifying biomarkers which can potentially serve as advanced diagnostics in tSCI. We will first provide an overview of current predictive models based on clinical evaluation and then continue describing biomarkers derived from neuroimaging, blood and CSF concentrations of several compounds, and the value of neurophysiologic readouts.

## Clinical Outcome Measures

Trying to predict recovery based on clinical parameters, a prognostic model was formulated including the five variables: age (<65 years vs ≥65 years), L3 and S1 motor, and light touch scores, which were acquired within 15 days post injury. It serves to predict independent walking 1-year post injury [12]. This model was simplified having only the three remaining variables: age (<65 years vs ≥65 years), L3 motor score, and S1 light touch score [13]. Critical analysis of both models with different patient cohorts revealed that these models achieved high prognostic accuracy for combined AIS categories, whereas applying these models to single AIS sub-groups led to considerably lower accuracy, thus limiting their applicability [14•, 15]. Models that include additional parameters or use machine learning algorithms have been developed but showed comparable or inferior predictive accuracy of patient’s mobility [16–19].

A recent approach for classifying a patient population into more homogeneous sub-groups is the unbiased recursive partitioning technique called conditional inference tree (URP-CTREE) that defines simple decision rules for partitioning the population [20]. This model was used for sub-classifying cervical sensorimotor complete (AIS A) SCI patients into sub-groups based on clinical parameters obtained within the first 2 weeks after injury [21]. The patients within one of these newly defined sub-groups expressed a more homogeneous recovery pattern. These results suggest that the use of such regression tree algorithms provides an easy method for partitioning a heterogeneous SCI patient population into homogenous sub-groups for improving prognostication [22, 23].

## Neuroimaging Biomarkers

Radiologic examinations are an integral part of the diagnostic evaluation following SCI and play an important role in assessing the level and severity of injury. Computed tomography (CT) provides excellent visualization of osseous anatomy and fractures and allows fast image acquisition [24]. However, its lack of soft tissue contrast and the insensitivity for visualization of neural damage might lead to underestimation of canal compromise and misdiagnosis in the clinical setting [25, 26]. In contrast to CT, conventional magnetic resonance imaging (MRI) helps to evaluate the damage to discoligamentous and neural structures after tSCI. It improves clinical decision-making early after trauma and facilitates finding the appropriate treatment [27]. The following paragraphs discuss both imaging parameters derived from conventional neuroimaging and advanced neuroimaging protocols that provide further insights into microstructural alterations.

## Conventional MRI at Lesion Site

**Figure 1.**
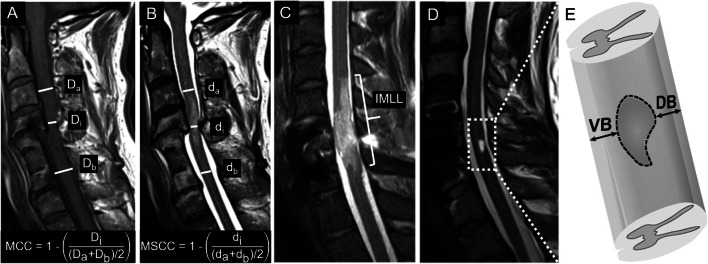
Lesion characteristics derived from standard MRI sequences. **A** Mid-sagittal T1-weighted MR image with the a-p diameters of the spinal canal at injury site (D_i_) and at nearest normal levels above (D_a_) and below (D_b_) the lesion, including the equation for MCC. **B** Mid-sagittal T2-weighted MR image with the a-p diameters of spinal cord at maximum injury site (d_i_) and at nearest normal levels above (d_a_) and below (d_b_) the lesion, including the equation for MSCC. **C** IMLL measured on a mid-sagittal T2-weighted MR image as the rostrocaudal length of the T2w hyperintense lesion. **D** Mid-sagittal T2-weighted MR image with a T2w hyperintense lesion for the determination of ventral and dorsal tissue bridges as indicated. Modified from Freund et al. [29]

The standard protocol in clinical routine after SCI comprises T1-weighted (T1w) and T2-weighted (T2w) MR images of the lesion level [24]. It serves to rapidly screen patients and represents an important prognostic indicator in the current clinical routine [28]. These sequences allow to measure several lesion characteristics such as maximum canal compromise (MCC), maximum spinal cord compression (MSCC), and intramedullary lesion length (IMLL) (see Fig. 1A–C for illustration). The MCC is calculated as the ratio of the anteroposterior (a-p) diameter of the spinal canal at the level of maximum injury (D_i_) to the a-p diameter at the nearest normal levels (D_a_ and D_b_) as measured on mid-sagittal T1w images. Similarly, the MSCC is derived as the ratio of the a-p spinal cord diameter at the level of maximum injury (d_i_) to the a-p diameter at the nearest normal levels (d_a_ and d_b_), measured on mid-sagittal T2w images [26]. Some studies demonstrated high inter- and intra-observer reliability of the MCC and MSCC measurements and correlation with injury severity and neurologic outcome [25, 26]. However, their predictive value disappeared, when the initial neurological status was available, demonstrating the limits of prognostication based on these measures [30, 31].

The IMLL, measured as the length from the most rostral to the most caudal apices of hyperintensive signal changes within the spinal cord on mid-sagittal T2w images, was found to be a good measure for assessing the severity of SCI and showed greater predictive value when compared to MCC and MSCC [32, 33••]. Despite this superiority, its clinical benefit is limited when the initial AIS grade is available, suggesting that the neurological status is more important to predict neurological outcome [31].

An alternative to these measures is the Brain and Spinal Injury Center (BASIC) score, which was proposed to improve the assessment of injury severity prognostication [34]. This classification system qualitatively grades the extent of intramedullary T2w signal abnormalities in the axial plane on a 5-point classification scheme. It was demonstrated that the BASIC score is a superior predictor of AIS grade compared to MCC, MSCC, and IMLL, and prognostication could be improved by integrating the clinical status and BASIC score [30, 33••, 35].

Important points to consider when assessing the lesion severity on conventional MR images are the exact anatomical location and local extent of the lesion, as lesions of similar size but different orientations can cause a diverse range and pattern of spared fiber tracts around the lesion [36]. An approach to include information about spared axonal fibers is measuring both ventral and dorsal tissue bridges on a mid-sagittal slice of a T2w image of the spinal cord (see Fig. 1D for illustration). The extent of preserved ventral and dorsal tissue paralleled patients’ recovery and electrophysiological recordings in several studies and was significantly related to patients’ walking ability [37–39]. Moreover, the width assessed at 1 month after injury was significantly associated with long-term neurological and neurophysiological outcome and could provide a more reliable prognostic measure [37, 38, 40••, 41]. In particular, the distinction between tract-specific impairment could serve as motor- and sensory-specific predictors separately, as ventral tissue bridges were significantly related to motor function and dorsal tissue bridges to sensory function [40••]. The assessment of tract-specific spinal cord damage on axial MRI slices might add valuable prognostic information in the future as well [42].

However, the T2w signal changes are not specific to the underlying pathophysiology and could represent both transient and irreversible pathologies. Furthermore, there is considerable variation over time and patients, and their quantification relies strongly on the subjective interpretation by the examiner [29, 31]. This highlights the need for further validation of measures derived from conventional MR imaging in the future in order to evaluate their diagnostic and prognostic value.

## Advanced Neuroimaging Markers

In contrast to conventional MRI sequences, which allow macrostructural assessment of neural tissue damage, new imaging protocols aiming at the quantification of specific tissue parameters serve to assess changes at the microstructural level [43, 44].

A prospective quantitative imaging modality that could serve as diagnostic and prognostic measure after SCI is diffusion tensor imaging (DTI). DTI assesses the microstructural integrity of fiber tracts and, thus, has higher sensitivity to early structural changes [28, 45]. Measuring spared white matter by means of non-invasive DTI as well as in post-mortem histological samples showed a strong correlation between both measures, pointing at the usefulness of DTI for assessing neuronal tract integrity non-invasively [46]. In both preclinical animal models of SCI and human SCI patients, differences in DTI parameters between SCI and healthy control groups were detected. In particular, SCI patients exhibited increased values of mean diffusivity (MD), indicating disorganization within the fiber tracts and decreased values of fractional anisotropy (FA), representing reduced axonal count and myelin content, at the level of injury [45, 47, 48]. Differences in these parameters between healthy controls and SCI patients were also found at levels above and below the lesion [49–51]. Furthermore, several studies showed significant associations between DTI measures and the functional outcome, pointing out the prognostic potential of this imaging technique [45, 48–53]. Contrasting these findings, one recent study comparing DTI and conventional MRI parameters could not prove superiority of DTI measures in predicting neurological outcome, although this study only comprised a small patient cohort [54].

Quantitative parameters for the assessment of microstructural changes are magnetization transfer saturation (MTsat) and longitudinal and effective transverse relaxation rates (R1 = 1/T1, R2* = 1/T2*) as implemented in the multi-parameter mapping protocol (MPM) [55]. These quantitative parameters are closely associated with myelin (MTsat, R1) and iron content (R2*) and thereby provide additional pathophysiologic insights into processes following spinal trauma as well as alternative advanced measures for tracking alterations after SCI [56–59]. It could be demonstrated in primates that quantitative MTsat is a robust parameter for tracking demyelination and loss of macromolecules after SCI [60]. Applied to SCI, MPM-based readouts could demonstrate reductions of myelin-sensitive parameters and increase in iron content in areas undergoing atrophy [61–64]. This is suggestive of demyelination and iron deposition within these atrophying areas. Changes in myelination and iron deposition were not only restricted to the proximity of the lesion site but also affected remote areas in both the spinal cord and the brain. Moreover, it could be demonstrated that these changes were correlated with long-term neurological outcome, speaking to their potential as new biomarkers for assessing injury severity and predicting outcome in SCI patients [61–63].

In summary, recent developments of MRI sequences allow for the assessment of microstructural neuronal integrity and pathophysiological alterations in myelination and iron deposition and might add valuable information for clinical decision-making and outcome prediction. Future studies are necessary for validating and implementing these methods into clinical practice.

## Markers in Cerebrospinal Fluid and Blood Serum

SCI usually leads to the disruption of the blood spinal cord barrier, resulting in the leakage of several neural tissue components into cerebrospinal fluid (CSF) and blood. Additionally, during the pathophysiological events following SCI, each stage is characterized by up- and downregulations of specific proteins, which renders them an optimal target as biomarker for tracking the stages after SCI [65]. Particularly, assessing the injury severity of unresponsive patients by blood- or CSF-derived markers might facilitate diagnostic workup [66]. Due to the plethora of different compounds, we focus in this review on the most promising factors, whose diagnostic and prognostic usefulness could be replicated (Table 1).

**Table 1.** Summary of spinal cord injury serum/CSF biomarkers

Biomarker	Pathophysiological process/origin	Main findings and diagnostic/prognostic utility	Reference
GFAP	Glial cell injury	Discrimination between AIS sub-groups by concentrations during early phase after SCI. Lower levels associated with better neurological recovery	Dalkilic et al, 2018 [10•], Kwon et al, 2017 [67], Ahadi et al, 2015 [68], Yang et al, 2018 [69]
S-100β	Glial cell injury	Discrimination between AIS sub-groups by concentrations during early phase after SCI. Lower levels associated with better neurological recovery	Dalkilic et al, 2018 [10•], Kwon et al, 2017 [67], Du et al, 2018 [70], Yang et al, 2018 [69]
NF-L, NF-H	Axonal injury	Higher levels in SCI patients compared to controls during acute phase. NF-L was correlated with long-term outcome, while NF-H showed no significant associations	Ahadi et al, 2015 [68], Yang et al, 2018 [69], Kuhle et al, 2015 [71], Casha et al, 2018 [72]
Tau	Neuronal injury	Elevated levels during acute phase after SCI and discrimination between AIS sub-groups. Levels correlated with long-term functional outcome	Dalkilic et al, 2018 [10•], Kwon et al, 2017 [67], Tang et al, 2019 [73], Caprelli et al, 2018 [74]
NSE	Neuronal cell body injury	Elevated levels in SCI patients without differences between AIS sub-groups. Inconsistent findings regarding predictive utility	Ahadi et al, 2015 [68], Du et al, 2018 [70], Li et al, 2014 [75], de Mello Rieder et al, 2019 [76]
IL-6, IL-8	Inflammation	Increased levels during acute and subacute phase. Predictive of AIS conversion in patients	Dalkilic et al, 2018 [10•], Kwon et al, 2017 [67], Yang et al, 2018 [69], de Mello Rieder et al, 2019 [76]
MCP-1	Inflammation	Elevated early after SCI. Results about prognostic utility are inconsistent	Dalkilic et al, 2018 [10•], Kwon et al, 2017 [67], Casha et al, 2018 [72], Heller et al, 2017 [77]
Albumin	Hepatic synthesis	Hypoalbuminemia after SCI was associated with poor long-term neurological outcome	Tong et al, 2018 [78], Vo et al, 2020 [79]
microRNAs	Various	Severity-dependent distinct changes in the expression profile after SCI with up- and downregulation of certain microRNAs	Sun et al, 2018 [80], Ding et al, 2020 [81], Park et al, 2019 [82], Li et al, 2019 [83], Tigchelaar et al, 2019 [84]

Overview of serum/CSF biomarkers with their pathophysiological origin and a short summary of their diagnostic/prognostic utility after SCI

These CSF based biomarkers can broadly be categorized into structural and inflammatory factors, as well as markers measured in routine blood analysis [85, 86]. Recent research focuses on the usefulness of microRNAs, an abundant class of small non-coding RNAs that were identified as tissue-specific markers of injury. These four classes of biomarkers will be discussed in the subsequent sections.

## Structural CSF/Serum Biomarkers

Structural biomarkers are mostly cell-specific proteins from neuronal tissue that leak into CSF and blood after trauma. These tissue-specific proteins are produced by different cells such as neurons or glia cells. Changes in the blood and CSF concentrations of several of these proteins following SCI were observed and are described in the following section.

Glial fibrillary acidic protein (GFAP) and calcium-binding protein S-100β are mainly derived from glial cells and were repeatedly shown to be elevated in both CSF and blood serum in the early acute phase after SCI in humans and animal models [10•, 67–70]. The levels of both proteins differed significantly between patients with different AIS grades, where most severely affected AIS-A patients expressed the highest measured levels. Additional to the differences between initial AIS categories, patients with lower protein levels exhibited a better neurological recovery than those patients with higher levels. These findings speak to their diagnostic and prognostic potential to assess the severity in the early phase of SCI [10•, 67, 68, 70].

Neurofilaments are abundant axonal cytoskeletal proteins that can be divided into three subunits, light (NF-L), middle (NF-M), and heavy chain neurofilament (NF-H), of which NF-L and NF-H have been studied most widely as potential biomarkers in SCI [87]. CSF and serum levels of both NF-H and NF-L were significantly higher in SCI patients compared to controls during the acute phase after SCI [68, 69, 71, 72]. However, the discrimination between individual AIS categories was not possible. Regarding the predictive potential, NF-L concentrations correlated with long-term motor outcome, whereas no significant associations between NF-H levels and functional outcome were found [71, 72].

Another protein that is expressed at high levels within neurons is Tau. It has an important function as stabilizer of microtubules and has been studied extensively in the context of Alzheimer’s disease due to its involvement in pathogenesis [88]. Increased extracellular levels might be indicative of neuronal damage; thus Tau could serve as a potential biomarker after SCI. In human SCI patients and animal models of SCI, Tau was elevated both in the blood and CSF during the acute phase following SCI with the concentration being related to injury severity and allowing to discriminate between AIS categories [10•, 67, 73, 74]. Moreover, the levels correlated with long-term functional outcome pointing at its predictive utility [10•, 67].

Neuron specific enolase (NSE), an enzyme that is mainly located in neuronal cytoplasm, was identified as a biomarker for neuronal damage in different pathologies [89]. NSE expression in neuronal tissue was shown to be upregulated in SCI models at the injury site [75]. Increased NSE concentrations in the serum were measured in SCI patients during the first days after trauma, although a clear categorization into individual AIS sub-groups was not possible [68, 70, 76]. Regarding its predictive utility, significantly lower concentrations in patients that had a better functional long-term outcome than in patients with only minor functional improvements were found [70]. Contrasting these findings, another study did not detect a strong correlation with long-term functional outcome [76].

## Inflammatory CSF/Serum Biomarkers

Markers of inflammation are upregulated and secreted by various cell types due to the neuro-inflammatory processes elicited by the trauma that may further worsen the injury. Therefore, they do not represent specific predictors of neuronal damage but rather reflect the general inflammatory reaction [85]. This section discusses the potential of some of these inflammatory cytokines as novel diagnostic measures after SCI.

Interleukins (IL) are a large family of cytokines having a diverse range of functions in immune response. Of these, particularly the pro-inflammatory IL-6 and IL-8 have been studied in SCI and seem the most promising biomarkers. IL-6 was reported to be elevated during the acute and subacute phase of SCI in a severity-dependent manner [10•, 67, 76]. Moreover, patients experiencing AIS improvement had significantly lower levels than patients not improving indicating its usefulness for prognostication [10•, 67]. Likewise, IL-8 was predictive of AIS conversion in the same manner, although IL-6 showed better performance [10•, 67].

Monocyte chemoattractant protein-1 (MCP-1) is a chemotactic cytokine (chemokine) produced by various cell types and plays an integral role for immune response [90]. It was found to be elevated in CSF and serum during the early acute phase of SCI and was predictive of neurological recovery [10•, 67, 77]. Contrasting these findings, Casha et al., although measuring an early increase in MCP-1 in CSF after SCI as well, did not detect significant correlations with neurological recovery [72].

Similar to structural CSF and serum biomarkers, some markers of inflammation seem to have the potential of improving the assessment of SCI patients. However, it must be noted that these factors only represent the general inflammatory reaction and are not specific to SCI injury.

## Markers in Routine Hematology

Investigating the utility of routinely measured blood parameters such as blood albumin levels for the assessment of injury severity and outcome prediction, it could be shown that hypoalbuminemia was associated with poor long-term neurological outcome and thus could serve as a marker for prognostication [78, 79]. Additionally, albumin levels measured during the subacute phase were significantly lower in the most severely affected AIS-A patients compared to other AIS groups, pointing at its utility in clinical risk assessment.

Two studies included a plethora of blood parameters measured during the first 2 weeks after SCI into predictive models and tested their contribution to improve the model [91•, 92]. Initial neurological function assessed by clinical evaluation was the most powerful predictor. However, blood measures of liver and kidney function, inflammation, and complete blood count (as marker for blood loss) could add significant prognostic value. These results suggest that prognostication might be improved by including regularly measured blood analytes as surrogates for general body function and secondary organ complications [93–95].

## MicroRNAs

MicroRNAs, a class of short non-coding RNAs, have drawn a particular interest during recent years as they are involved in several regulatory processes including processes after SCI such as regulation of post-injury inflammation, neuroplasticity, and axon and neuron regeneration [96, 97]. Following SCI, some of these microRNAs are upregulated leading to a decrease in expression of their target genes while others are downregulated. This in turn leads to a change in the microRNA expression profile and thereby to a different measurable spectrum of microRNAs in blood and CSF [80, 81]. This expression profile varies over time after SCI providing the possibility of characterizing each post-injury stage [82, 83]. A recent study that measured microRNA profiles in CSF and serum of SCI patients showed a severity-dependent expression profile in CSF with AIS-A patients expressing highest concentrations of total microRNAs in CSF shortly after trauma [84]. The extent of up- and downregulation of specific microRNAs in CSF varied between different AIS groups as well. Most severely affected patients expressed highest up- and downregulations pointing at the diagnostic utility of microRNAs. Using a distinct set of these up- and downregulated microRNAs, AIS grade improvement could be predicted for patients that were classified as AIS-A at baseline.

## Neurophysiologic Markers

Neurophysiologic techniques, such as measuring nerve conduction, motor-evoked potential (MEP), and somatosensory-evoked potentials (SEP), provide objective measures of neuronal integrity and allow the differentiation between demyelination and axonal damage [98••, 99]. Their value as independent tool for stratifying SCI patients into sub-groups and their predictive utility were already demonstrated and validated several years ago [99–102]. However, some questioned whether these electrophysiological parameters could add valuable information for improving functional outcome prediction. Hupp et al. could show in a multicenter study that including electrophysiological multimodal parameters into the prediction model leads to better prediction precision of this model, even if the clinical neurological status is available [98••]. These results suggest that the assessment of neurologic function and prognostic accuracy in SCI patients can be improved by adding neurophysiological methods to standardized clinical evaluation. Nonetheless, this study also identified total motor score as the best single prediction parameter, which once more highlights the importance of clinical evaluation during the diagnostic workup for the assessment of severity in SCI.

## Conclusion

This review described the latest progress in identifying reliable biomarkers for traumatic SCI and improving predictive models. Clinical evaluation by standardized neurological examination constitutes the gold standard for assessing injury severity and predicting functional outcome. Nevertheless, these models can be improved by including advanced diagnostics, suggesting that a multimodal approach—including neuroimaging and CSF/blood markers—improves the accuracy of predicting individual trajectories of recovery. Future studies investigating the exact potential of this approach with multivariate models that can accommodate multimodal data are required for demonstrating the utility of combinations of these advanced biomarkers.
